# Non-Contact Laser Ultrasound Detection of Internal Gas Defects in Lithium-Ion Batteries

**DOI:** 10.3390/s25072033

**Published:** 2025-03-25

**Authors:** Dongxia Tang, Chenguang Xu, Guidong Xu, Sen Cui, Sai Zhang

**Affiliations:** Institute of Ultrasonic Testing, Jiangsu University, Zhenjiang 212013, China; 2212226018@stmail.ujs.edu.cn (D.T.); chenguang_x@hotmail.com (C.X.); xugd@ujs.edu.cn (G.X.); scui@ujs.edu.cn (S.C.)

**Keywords:** lithium-ion battery, defect detection, laser ultrasonic, non-destructive testing

## Abstract

Non-contact laser ultrasonic detection technology provides an innovative solution for evaluating the internal conditions of lithium-ion batteries (LIBs), offering significant advantages in gas defect assessment and structural defect identification. This study proposes a method for evaluating internal gas defects in LIBs based on a non-contact laser ultrasonic system. The system uses a pulsed laser to generate ultrasonic waves, with a full-optical probe receiving the signals, enabling high-resolution imaging of the internal features of the battery. The study analyzes key ultrasonic characteristics under different laser parameters (energy, pulse width, and focal length) and their correlation with defective regions. Through both time-domain and frequency-domain analysis of the ultrasonic features, the results demonstrate that the signal amplitude attenuation characteristics of ultrasound in media with acoustic impedance mismatches can be used for precise detection and quantitative characterization of gas defect regions within the battery. This non-contact technology offers a promising method for real-time, non-destructive monitoring of the internal condition of lithium-ion batteries, significantly enhancing battery safety and reliability.

## 1. Introduction

The demand for lithium-ion batteries (LIBs) has surged in recent years, driven by their widespread application in portable electronics, electric vehicles, and renewable energy storage [1,2,3,4]. From portable electronics and electric vehicles to large-scale renewable energy storage, LIBs power a vast range of applications due to their high energy density, longevity, and relatively lightweight structure [5,6,7]. The condition of porosity defects within battery cells is a critical factor affecting the efficiency and safety of LIBs [8]. Porosity defects in LIBs arise from various factors, including uneven electrolyte distribution, gas release during charge and discharge cycles, thermal expansion or contraction-induced material cracking, and degradation or poor interfacial bonding [9]. These defects can significantly affect the battery’s structural integrity and performance, potentially leading to safety hazards. Consequently, the effective detection and assessment of porosity defects are of paramount importance.

The commonly used methods for detecting pore defects in LIBs include direct observation, spectroscopic analysis, and non-destructive imaging techniques. The direct observation method can intuitively determine whether a battery exhibits swelling or bulging, but it is challenging to detect the generation of trace amounts of gas with this approach. Spectroscopic analysis methods include gas chromatography and mass spectrometry [10], online electrochemical mass spectrometry (OEMS) [11], and Raman spectroscopy [12,13,14], among others. These methods are capable of effectively analyzing the composition and concentration of gases. However, the process of transferring the internal gases of the battery to the spectroscopic analysis equipment may lead to structural damage and potentially impact the battery’s performance. Non-destructive imaging methods comprise ultrasonic testing, X-ray inspection [15,16,17], and neutron imaging [18]. X-ray inspection technology can indirectly identify gases by monitoring structural deformations within the battery, although its visualization capabilities are significantly limited, making it difficult to sensitively and accurately capture the dynamic evolution of internal gases. Neutron imaging technology utilizes the interaction mechanisms between neutrons and atomic nuclei to effectively identify the distribution of gas generation in various regions within the battery. However, this technique is constrained by high equipment costs, significantly limiting its application scope.

Ultrasonic testing has gained significant attention in recent years as an effective non-destructive evaluation technique in the study of battery materials [19,20,21,22]. Common non-contact ultrasonic inspection techniques include air-coupled ultrasound [23], laser ultrasound [24], and electromagnetic acoustic transducers (EMATs) [25]. EMATs are more suitable for field applications but are limited to detecting defects near the probe. Air-coupled ultrasound enables remote inspection but is constrained by impedance mismatch and frequency range limitations. Laser ultrasound, which excites and detects ultrasonic waves in a non-contact manner, offers high spatial resolution, a broadband response, and applicability to non-conductive materials, effectively overcoming the limitations of EMATs and air-coupled methods. Therefore, laser ultrasound demonstrates significant advantages in detecting internal defects in LIBs.

Laser ultrasonic technology utilizes pulsed lasers to generate ultrasonic waves through thermoelastic expansion, enabling defect detection without the need for coupling media. Several studies have investigated ultrasonic methods for LIB defect detection, focusing on different aspects. Bruder et al. [26] demonstrated that laser ultrasound can identify defects by detecting reflections along the wave propagation path. Choi et al. [27] developed a laser ultrasonic detection system for identifying welding defects in LIBs. Sampath et al. [28] utilized a non-contact laser ultrasound system for real-time monitoring of LIB status during charging and discharging. While these studies demonstrate the potential of ultrasonic techniques in LIB inspection, existing research has primarily focused on structural defects or battery state monitoring. However, internal gas defects, which pose significant risks to battery safety and performance, remain challenging to detect using conventional ultrasonic methods due to their weak acoustic contrast with surrounding materials.

This study presents a non-contact method based on laser ultrasound technology for evaluating internal gas defects in LIBs. The laser ultrasonic system consists of a pulsed laser that generates ultrasonic waves and an optical microphone that receives the acoustic signals. By analyzing the attenuation characteristics of ultrasonic waves within the battery, the internal gas defects are assessed. The main contributions of this study include (1) the application of a fully non-contact laser ultrasonic technique for detecting internal gas defects in LIBs; (2) the realization of real-time monitoring and assessment of internal defects in batteries; and (3) the validation of the feasibility and accuracy of laser ultrasound technology for defect size measurement.

The rest of the paper is organized as follows. Section 2 discusses the generation mechanism of laser ultrasound and the propagation characteristics of ultrasonic waves. Section 3 presents the experimental setup for the non-contact laser ultrasonic testing of LIBs, including data acquisition parameters, scanning procedures, and the relationship between ultrasonic properties and gas defects within the battery. Section 4 provides the experimental results and discussions, comparing and analyzing the effects of different laser ultrasonic scanning parameters and imaging outcomes. Finally, Section 5 concludes the study and summarizes the findings.

## 2. Theory

### 2.1. Laser-Induced Ultrasound Excitation Theory

The excitation mechanism of laser-generated ultrasonic signal comprises thermoelastic excitation and ablation. When the pulsed laser energy is higher than the cell damage threshold, the cell surface temperature rises rapidly to the melting point of the cell, resulting in melting of the cell and formation of plasma, then a part of the cell surface will be ejected, and a reaction force will be applied to the cell surface, and a compression pulse will be generated. When the pulsed laser power density is lower than the damage threshold of the cell, part of the laser energy incident on the sample surface is absorbed by the surface layer and converted into heat energy, and a transient temperature field with a gradient distribution is formed near the laser light source due to the absorbed heat on the cell surface. The thermal expansion of the cell due to uneven heating produces tangential pressure on the surface and excites shear waves, longitudinal waves and surface waves. In the thermoelastic mechanism, the pulse laser energy is lower than the damage threshold of the material [29], so the damage to the cell is avoided.

Laser radiation on the surface of cell has a Gaussian distribution, and the thermal diffusion equation under thermoelastic mechanism can be expressed as [30]

(1) $$\rho c_{v}\frac{\partial T}{\partial t}=\nabla \cdot \left(k\nabla T\right)+Q,$$

where ρ is the material density, $c_{v}$ represent the constant pressure heat capacity of the material, *T* is the temperature distribution function, *k* is thermal conductivity, and *Q* denotes the power density of the heat source generated by laser source. The energy transfer inside the battery materials is completed in the form of heat conduction. Assuming that the laser beam is completely absorbed at the surface within a small volume, the effects of thermal convection and thermal radiation on the temperature change can be neglected. Thus, the heat source *Q* due to the penetration of the incident laser beam is given by

(2) $$Q\left(x,t\right)=I_{0}\left(1-R\right)f\left(x\right)g\left(t\right),$$

where $I_{0}$ represent the peak power density of the incident laser, and *R* is the reflection coefficient of the cell surface. f(x) and g(t) are the spatial and temporal distributions of laser pulses, respectively, and can be described as

(3) $$\begin{array}{cc} \; & f\left(x\right)=\exp\left(-\frac{{\left(x-x_{0}\right)}^{2}}{r_{0}}\right), \end{array}$$

(4) $$\begin{array}{cc} \; & g\left(t\right)=\frac{t}{t_{0}}\exp\left(-\frac{t}{t_{0}}\right), \end{array}$$

where $x_{0}$ and $r_{0}$ denote the initial position and spot radius of the pulsed incident laser, and $t_{0}$ is the pulse width.

The temperature change around the heat source on the battery surface exhibits a gradient distribution and produces thermal stress, which excites ultrasonic waves and forms a displacement field. In this process, the ultrasound propagation in the thermoelastic material follows the Navier–Stokes equations

(5) $$\left(\lambda -2\mu \right)\nabla \left(\nabla \cdot U\right)-\mu \nabla \times \nabla \times U-\alpha \left(3\lambda +2\mu \right)\nabla T=\rho \frac{\partial^{2}U}{\partial t^{2}},$$

where λ and μ are the Lamé constants, α is the coefficient of thermal expansion, and *U* denotes the instantaneous displacement.

### 2.2. Principle of Ultrasonic Testing

LIBs are composed of porous media in which ultrasonic waves experience attenuation due to acoustic impedance mismatch during propagation. The primary attenuation mechanisms for acoustic waves transmitted within lithium-ion batteries include absorption by the medium, scattering due to heterogeneous structures, interfacial losses at material boundaries, and diffusion-induced energy dissipation. Assume there is a point in the ultrasonic sound field, where the sound pressure is represented as:

(6) $$P=-A\rho c\cdot \sin(\omega t_{0}-\frac{x}{c})=\rho cv,$$

where *A* represents the amplitude of the particle at that point, ω indicates the angular frequency at that point, *v* refers to the vibration velocity of the particle, *x* is the distance between the particle and the wave source, and *c* is the sound velocity of the ultrasonic wave in the medium. The acoustic energy passing through a unit area perpendicular to the direction of ultrasonic wave propagation per unit time in the ultrasonic sound field is given by:

(7) $$I=\frac{1}{2}\rho cA^{2}\omega^{2}=\frac{1}{2}\frac{P^{2}}{\rho c}.$$

Acoustic impedance is represented as:

(8) Z=ρc.

When sound waves propagate through a medium and encounter a physical interface with an impedance mismatch, reflection and transmission occur. The pressure transmissivity (t) and reflectivity (r) can be expressed as:

(9) $$T=\frac{\frac{P_{t}^{2}}{2Z_{2}^{2}}}{\frac{P_{0}^{2}}{2Z_{1}}}=\frac{4Z_{1}Z_{2}}{{\left(Z_{2}+Z_{1}\right)}^{2}},$$

and

(10) $$R=\frac{\frac{P_{r}^{2}}{2Z_{1}}}{\frac{P_{0}^{2}}{2Z_{2}}}=\frac{{\left(Z_{2}-Z_{1}\right)}^{2}}{{\left(Z_{2}+Z_{1}\right)}^{2}},$$

where $P_{0}$ represents the incident wave sound pressure, $P_{t}$ is the transmitted wave acoustic pressure at the interface, $P_{r}$ represents the reflected wave acoustic pressure at the interface, and $Z_{1}$ and $Z_{2}$ represent the acoustic impedances of the first medium and the second medium on either side of the interface, respectively.

**Figure 1:**
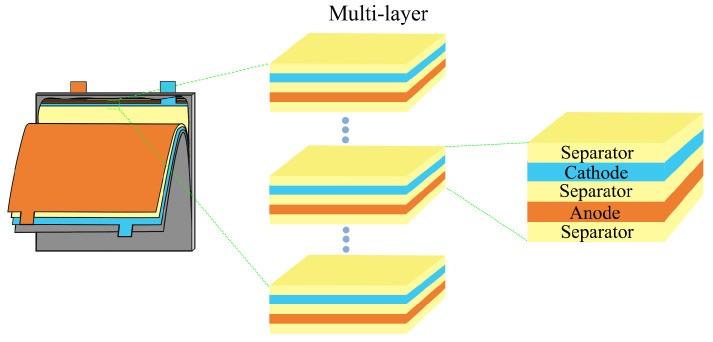
Schematic diagram of the multilayer structure of a lithium-ion battery.

**Figure 2:**
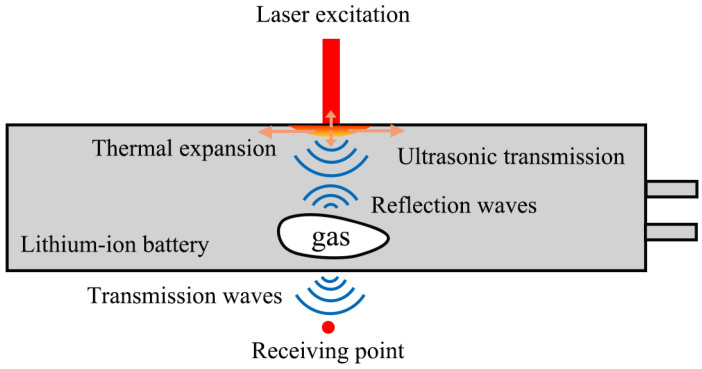
Photo-thermal ultrasound process in lithium-ion batteries with gas defects.

LIBs are designed with a periodic multi-layer structure, where key components such as active materials, electrolyte, and separator are precisely stacked together [31,32], as shown in Figure 1. From the structure of a LIB cell, it can be observed that its periodic stacking is composed of a basic unit cycle of “separator–negative electrode–separator–positive electrode”, with the last cycle unit ending in the absence of a positive electrode plate. The overall stacking can reach up to hundreds of layers. According to the principle of acoustic wave propagation, the ultrasound waves generated by laser excitation on the surface of the LIB will experience partial reflection and transmission when encountering porosity defects due to the mismatch of acoustic impedance between air and the battery material, as shown in Figure 2. This acoustic effect results in a significantly lower amplitude of the received ultrasonic signal in batteries containing porosity defects compared to defect-free batteries. This study utilizes the attenuation of ultrasonic waves in the electrolyte to establish an effective method for assessing internal porosity defects in LIBs.

## 3. Experimental Methodology

**Figure 3:**
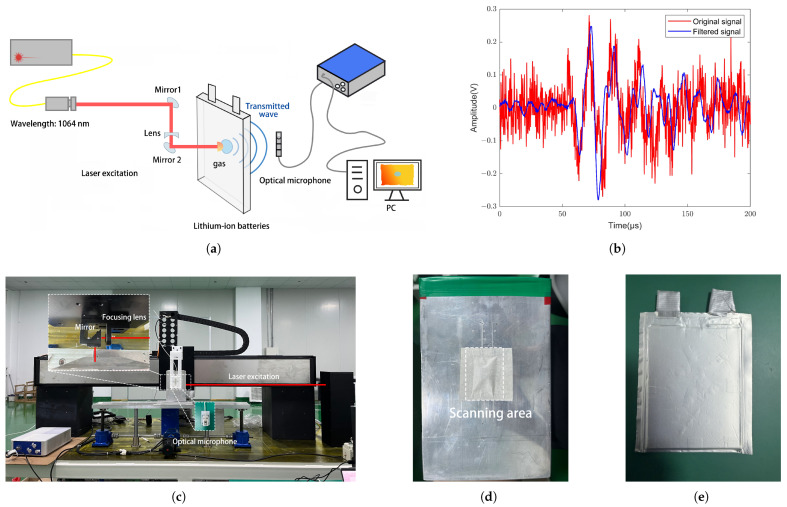
A non-contact laser ultrasonic system for estimating the internal air defects of lithium-ion batteries. (**a**) Schematic diagram of a laser ultrasound imaging system utilizing a SEAO probe. (**b**) Comparison of hardware-filtered signals. (**c**) Diagram of experimental setup. (**d**) Prismatic LIB with surface bubbles. (**e**) Pouch LIB with internal air defects.

Figure 3a shows the laser ultrasound imaging system equipped with a sensitivity-enhanced all-optical (SEAO) probe, which primarily consists of an excitation unit and a reception unit. In the excitation part, a pulsed laser (YDFLP-CL-500-50-W, JPT Opto-electronics, Shenzhen, China), which has a pulse width of 60 ns, a wavelength of 1064 nm, a repetition rate of 2 kHz, and a peak pulse energy of 50 mJ, is focused on the battery surface using a convex lens, resulting in a laser beam radius of approximately 2.5 mm at a focal distance of 20 mm. The emitted laser beam is focused onto the battery surface to generate ultrasonic waves, which propagate through the interior of the battery to the opposite side, where the signals are detected by the receiving unit consisting of an optical microphone (Eta450 Ultra, XARION Laser Acoustics, Wien, Austria). This directly measures the ultrasonic signals in the air by detecting changes in the refractive index as acoustic waves pass through a Fabry–Pérot etalon [33,34], with a sensitivity of 100 mV/Pa and a self-noise of 10 mPa across the full bandwidth. The optical microphone is secured using a rectangular clamp and positioned approximately 3 mm from the battery surface for through-transmission reception. This setup ensures stable movement during the scanning process while simultaneously receiving the signals. The received ultrasonic signals are converted into electrical signals by the optical microphone, which are then acquired and stored using a data acquisition card. Due to electromagnetic interference during the detection process, a Gaussian filter with a frequency range of 50 kHz to 130 kHz is applied to the received signals in MATLAB R2021a to improve the signal-to-noise ratio, as shown in Figure 3b.

In this study, two types of LIBs were selected to ensure a comprehensive evaluation of the proposed detection method. The first type is a prismatic LIB with artificial bubbles applied to its surface, which allows controlled simulation of gas defects. The second type is a pouch LIB that contains internal gas defects, providing a more realistic representation of gas-related issues that may occur during battery operation. The use of these two samples enables the validation of the laser ultrasonic detection technique under different types of gas defect scenarios, improving the reliability and applicability of the method for real-world battery defect detection. The prismatic cell has geometric dimensions of 200 mm × 130 mm × 36 mm, a nominal capacity of 100 Ah, and a nominal voltage of 3.2 V, with artificial bubbles on its surface measuring 40 mm × 25 mm, as shown in Figure 3d. The pouch lithium-ion battery used in this study contains internal gas defects that originated during the manufacturing process, likely due to reactions such as electrolyte decomposition or incomplete sealing. To ensure the gas defects are intrinsic to the battery, the sample was kept sealed and uncycled before testing, preventing external contamination. This approach allows for a controlled evaluation of the internal gas defects. The pouch LIB is a 1 Ah $LiFePO_{4}$ cell with dimensions of 104 mm × 85 mm × 10 mm, as shown in Figure 3e.

## 4. Results and Discussion

### 4.1. Study on System Parameters

Choosing an appropriate laser spot size is crucial for the accurate detection of internal gas defects in LIBs using laser ultrasound. The spot size directly influences the distribution of laser energy within the battery and the efficiency of ultrasonic wave excitation. A spot size that is too small results in highly concentrated laser energy, limiting the excitation region and making it challenging to accurately assess gas defects over a larger area. Conversely, an excessively large spot size leads to dispersed energy, weakening the ultrasonic signals and reducing sensitivity to small-scale gas defects. Due to the use of an infrared laser in this study, the laser spot was not directly observable. Therefore, optimizing the spot size ensures sufficient signal strength while covering an appropriate detection area, thereby enhancing the accuracy of internal gas defect analysis in batteries. The spot size was adjusted by varying the distance between the incident laser and the battery surface to optimize the laser parameters.

**Figure 4:**
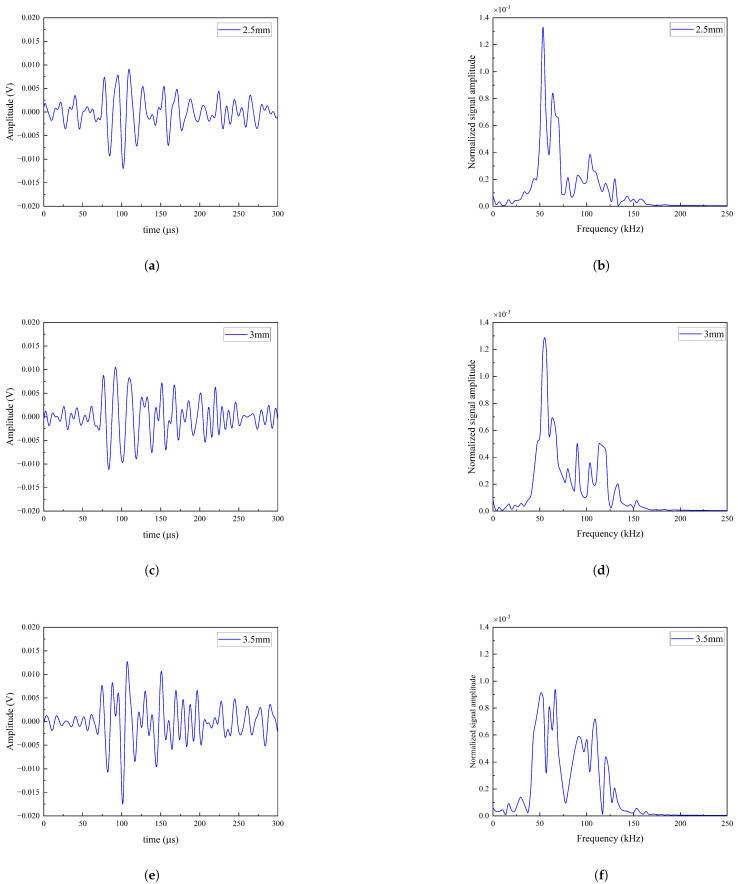
Effects of different laser spot sizes on the frequency characteristics and amplitude distribution of laser ultrasonic signals. Laser ultrasonic waveforms for spot sizes of (**a**) 2.5 mm, (**c**) 3 mm, and (**e**) 3.5 mm; corresponding frequency-domain representations for (**b**) 2.5 mm, (**d**) 3 mm, and (**f**) 3.5 mm.

When the spot diameter was 2.5 mm, as shown in Figure 4a, the signal displays relatively high amplitude with distinct and densely packed oscillations in the waveform. After Fourier transform processing, the corresponding frequency-domain representation was obtained, as shown in Figure 4b, with a center frequency of 53 kHz. The smaller spot size concentrated the signal energy, resulting in a prominent amplitude peak at this frequency, which corresponds to the higher signal amplitude and denser waveform oscillations.

As the spot size increased to 3 mm, as shown in Figure 4c, the signal amplitude further increased, while the waveform oscillations remained distinct but became less dense. The corresponding frequency-domain characteristics are shown in Figure 4d, wherein the center frequency shifted to 57 kHz, accompanied by multiple amplitude peaks and a broader energy distribution across the frequency spectrum. This indicates that the increase in spot size leads to energy dispersion, introducing additional frequency components in the spectrum while enhancing the overall signal amplitude.

When the spot size further increased to 3.5 mm, as shown in Figure 4e, the signal amplitude continued to rise, with the number of oscillations remaining substantial, yet the waveform density decreased further, resulting in a broader frequency distribution. Figure 4f shows that the center frequency reached 67 kHz, accompanied by multiple prominent amplitude peaks. Although the overall waveform characteristics measured at the three different spot sizes were similar, the variations in amplitude indicate that the spot radius significantly influences the battery’s response intensity. With an increasing spot radius, the frequency components became more complex, with multiple peaks distributed over a wider frequency range. This phenomenon highlights the critical importance of adjusting the spot size to optimize laser energy coupling and material response.

**Figure 5:**
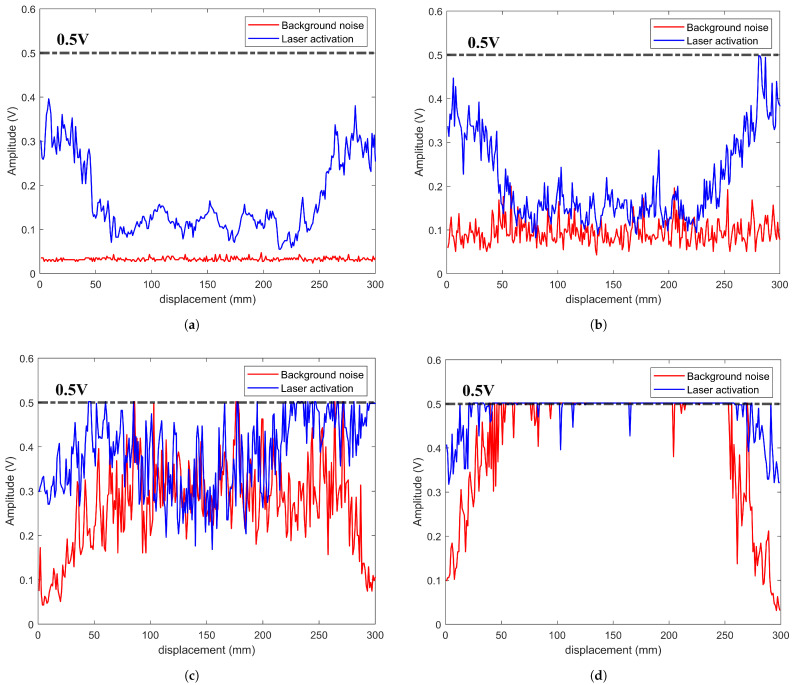
Fluctuations in background noise and laser loading signals at different scanning speeds. (**a**) 20 mm/s; (**b**) 500 mm/s; (**c**) 1000 mm/s; and (**d**) 1500 mm/s.

During the movement of the scanning device, mechanical vibrations and electromagnetic interference (EMI) from nearby electrical components can significantly affect the optical microphone’s performance, which is highly sensitive to such noise. To evaluate the impact of these factors, we varied the scanning speed of the laser ultrasonic imaging system and observed the effects on background noise and peak signals using a 0.5 V amplitude threshold, as shown in Figure 5.

At a scanning speed of 20 mm/s, the average background noise amplitude was 0.0324 V, while the average signal amplitude after laser application was 0.1134 V, as shown in Figure 5a. At 500 mm/s, these values increased to 0.0915 V and 0.1481 V, respectively, indicating greater noise interference at higher speeds. Further increasing the speed to 1000 mm/s and 1500 mm/s, as shown in Figure 5c,d, resulted in significant signal fluctuations, with over 50% of both background noise and laser-induced signals exceeding the 0.5 V threshold, making signal collection and imaging impossible.

Clearly, during the scanning process, the rigidity of the plastic fixture used by the optical microphone is insufficient, which limits the scanning speed. As the scanning speed increases, the amplitude of the scanning device’s vibrations also increases, leading to a greater impact from noise. Therefore, in order to ensure clear and stable battery scanning images when using the laser ultrasonic imaging system, this study controls the scanning speed to within 100 mm/s.

### 4.2. Laser Ultrasonic C-Scan Imaging

**Figure 6:**
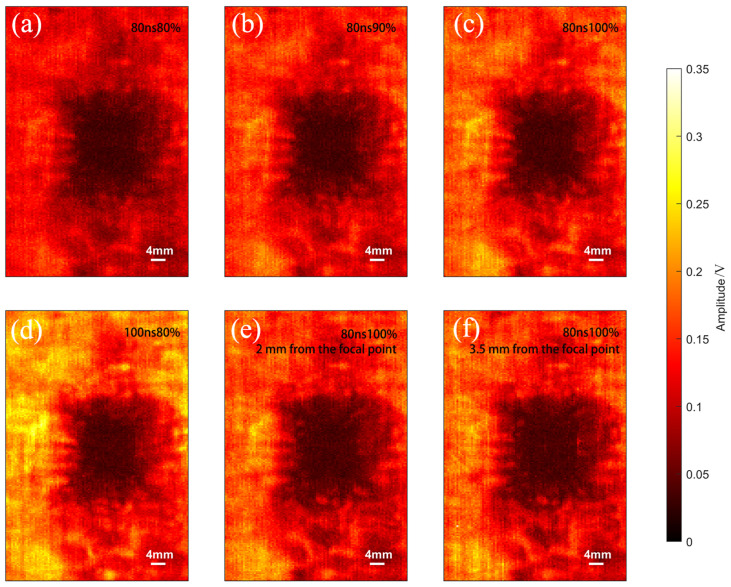
C-scan feature image of a LIB bubble with a width of 25 mm. (**a**) Laser power at 80% with a pulse width of 80 ns. (**b**) Laser power at 90% with a pulse width of 80 ns. (**c**) Laser power at 100% with a pulse width of 80 ns. (**d**) Laser power at 100% with a pulse width of 100 ns. (**e**) Laser power at 100% with a pulse width of 80 ns, 2 mm from the focal point. (**f**) Laser power at 100% with a pulse width of 80 ns, 3.5 mm from the focal point.

Different laser energy levels and pulse widths affect ultrasonic imaging results. Higher laser energy enhances ultrasonic wave amplitude, resulting in stronger signals and clearer images. However, excessive energy may damage the material or introduce additional noise. Pulse width, conversely, affects the frequency characteristics of ultrasonic waves. Shorter pulse widths produce higher-frequency signals, which improve spatial resolution but may reduce penetration depth. Conversely, longer pulse widths generate lower-frequency signals, making them suitable for deeper inspection at the expense of finer image details. In this study, a laser ultrasonic imaging system was used to scan LIBs containing bubbles. The scanned area, shown in Figure 3d, measured 50 mm × 100 mm. This research systematically examined the effects of various laser energy and pulse width combinations, as well as different spot sizes, on imaging results and bubble area identification. As shown in Figure 6, the near-black areas represent regions of amplitude attenuation, while the white and yellow areas indicate regions with better transmission.

**Figure 7:**
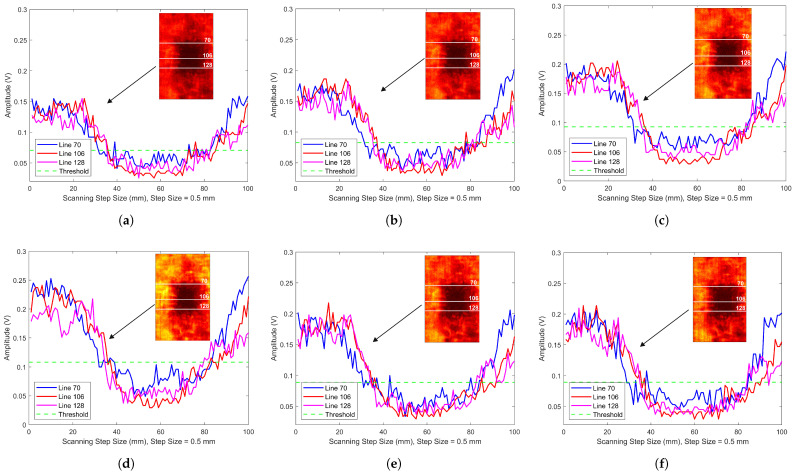
(**a**) Peak distribution of Figure 6a. (**b**) Peak distribution of Figure 6b. (**c**) Peak distribution of Figure 6c. (**d**) Peak distribution of Figure 6d. (**e**) Peak distribution of Figure 6e. (**f**) Peak distribution of Figure 6f.

The laser ultrasonic detection system was used to scan the LIB containing bubbles, with the pulse width fixed at 80 ns while varying the laser power. Figure 6a, b, and c show the scanning results at laser powers of 80%, 90%, and 100%, respectively. The imaging results reveal that variations in laser power do not significantly impact the overall imaging quality. However, as laser power increases, the overall amplitude of the waveform signal also rises. In the C-scan image analysis of samples with defects, a defect width evaluation method based on waveform peak values and a dynamic threshold was developed to quantify defect characteristics effectively under different laser parameters. First, a defect-free area far from the defect was selected from the image, and its average amplitude served as a baseline. A dynamic threshold, Th, set at 70% of this baseline, was used to identify defect regions falling below the threshold. Peak waveform data was then extracted from three parallel scan lines on the LIB, with defect regions identified using the dynamic threshold. The start and end positions of the defect in each row were determined to calculate each row’s defect width, which was converted to physical width. The average width $\bar{L}$ of the three scan lines represents the overall defect width, ensuring the reliability and stability of the results. The calculated values were then compared with the actual measured values $L_{0}$ to determine the relative error ε, as shown in Table 1. The widths $L_{1}$, $L_{2}$, and $L_{3}$ correspond to the values at the 70th, 106th, and 128th scan lines, respectively. The peak signal plots for the three scan lines in Figure 6a are shown in Figure 7a. When the pulse width is 80 ns and the laser power is set to 80%, the dynamic threshold Th is 0.071 V and $\bar{L}$ is 24.67 mm. Figure 7b shows the peak signal of the three scan lines in the C-scan image for a pulse width of 80 ns and laser power of 90%, where the calculated Th is 0.083 V and $\bar{L}$ is 24.83 mm. When the laser power increases to 100%, the calculated average width $\bar{L}$ remains 24.67 mm, while Th increases to 0.093 V, as shown in Figure 7c. By analyzing the dynamic threshold and defect width under different laser power settings, it can be observed that as the laser power increases, the dynamic threshold gradually rises, and the amplitude of the defect-free areas also increases. This observation is consistent with the phenomena seen in Figure 6. By fixing the pulse width at 80 ns and gradually increasing the laser power, the calculated defect width aligns with the actual measurement, with an error consistently below 2%, confirming the reliability and accuracy of the method under different laser parameters.

The effect of pulse width on laser ultrasonic imaging results is significant, as changes in pulse width directly influence signal resolution, imaging clarity, and detail representation. By adjusting the pulse width appropriately, energy transfer and signal reflection can be optimized, thereby enhancing the accuracy of defect detection and imaging quality. Figure 6d presents the imaging result at a pulse width of 100 ns and laser power of 100%. Compared to Figure 6c, the increased pulse width enhances the signal amplitude. Figure 7d shows the peak scan of the three rows under the same conditions, with a dynamic threshold Th of 0.108 V. The measured average bubble width $\bar{L}$ is 24.67 mm, consistent with the average width in Figure 7c at a pulse width of 80 ns and laser power of 100%, with an error of 1.32%. Therefore, under the same laser power, increasing the pulse width significantly enhances the signal amplitude, with minimal impact on the measured bubble width. This further validates the method’s stability and accuracy in evaluating defect width across different pulse widths.

The distance of laser incidence directly affects the concentration of laser energy and the quality of imaging. Figure 6e,f show the bubble scanning images at distances of 2 mm and 3.5 mm from the focal point. When the incident laser is positioned 2 mm from the focal point, the bubble C-scan image is as shown in Figure 6e, with the laser parameters consistent with those in Figure 6c. Notably, the black area surrounding the bubble in Figure 6e is larger than the corresponding area in Figure 6c. As shown in Figure 7e, when the incident laser is positioned 2 mm from the focal point, the calculated dynamic threshold Th is 0.089 V and the average bubble width is 28.33 mm, which shows a considerable deviation from the actual measurement, with an error of 13.32%. As the distance increases to 3.5 mm, the amplitude decreases and the black area expands further, as shown in Figure 6f. From Figure 7f, it can be observed that the dynamic threshold Th remains at 0.089 V and the average bubble width is 28.5 mm, with the error increasing to 14%. In conclusion, the distance of laser incidence significantly influences both the concentration of laser energy and the quality of imaging. As the distance from the focal point increases, the amplitude of the signal decreases, leading to a larger black area surrounding the bubble in the C-scan images. This results in a greater deviation in the calculated bubble width, demonstrating that precise control of the laser focus distance is crucial for accurate defect detection and measurement.

**Figure 8:**
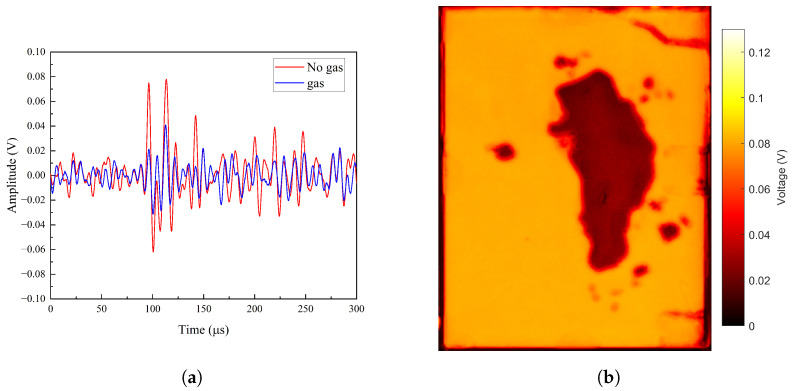
Results of laser ultrasonic detection for gas defects in LIBs. (**a**) Comparison of signal amplitudes for gas and non-gas defects. (**b**) Laser ultrasonic C-scan imaging.

During laser ultrasonic detection, ultrasonic waves passing through LIBs experience signal attenuation in regions with bubble defects. This occurs due to the differences in physical properties between areas with bubbles and those without, which affect the propagation speed and energy transfer of the ultrasonic waves within the medium. Bubbled regions create higher acoustic impedance mismatches, leading to partial reflection and scattering of ultrasonic waves, thus weakening the transmitted signal. By comparing the laser ultrasonic detection signals of LIBs with and without gas bubbles, it was found that the peak signal amplitude of the bubble-free region was 78 mV, while the peak signal amplitude of the gas-containing region was 40 mV higher, with a difference of 38 mV, as shown in Figure 8a. This attenuation characteristic can be leveraged to image the battery’s internal bubble defects. Ultrasonic scanning was performed on the pouch LIB with bubble defects, utilizing the acoustic impedance mismatch between different media. The scanning area was 100 mm × 80 mm.

Laser ultrasonic C-scan detection using the transmission method was performed on the LIB with gas defects, and imaging analysis of internal gas defects was conducted through amplitude difference imaging. The scan step size was 0.5 mm. The C-scan results are shown in Figure 8b, where the yellow region represents areas with higher ultrasonic penetration, and the blue region indicates areas with lower ultrasonic penetration. The results clearly reveal the size and spatial distribution characteristics of the gas defects inside the LIB. This suggests that the gas defect region causes scattering and mode conversion of the sound waves, resulting in more dispersed energy distribution. This further demonstrates that using laser ultrasonics in transmission mode to receive signals from the back of the LIB for gas defect detection is feasible.

**Figure 9:**
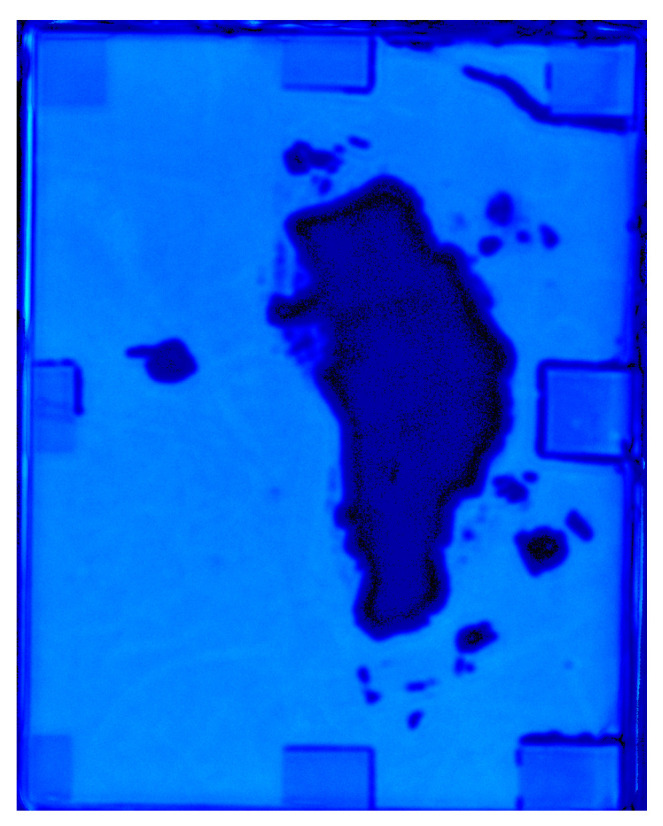
Scanning acoustic microscopy image of gas-containing defects.

To verify the defects identified in the two-dimensional image shown in Figure 8b, a 2D scanning image of the pouch LIB was obtained using an scanning acoustic microscope with a 2.5 MHz excitation frequency, as shown in Figure 9. By comparing the images obtained from laser ultrasonics and the acoustic microscope scan, it was observed that the defect areas coincided, further confirming the accuracy and reliability of the laser ultrasonic detection method for identifying internal defects in the LIB.

## 5. Conclusions

This study establishes the effectiveness of a non-contact laser ultrasonic detection system for evaluating internal gas defects in LIBs. By observing the noise interference level of laser ultrasonic signals at different scanning speeds, it is evident that both background noise and signal amplitude are affected as the scanning speed increases, with significant noise interference at higher speeds. Therefore, to achieve optimal performance during battery scanning and imaging with the laser ultrasonic system, the scanning speed must be maintained below 100 mm/s to ensure accurate signal acquisition and enhanced image quality. Additionally, adjustment of the spot size indicates that a smaller laser spot generates a concentrated energy response, exhibiting distinct oscillations and a prominent center frequency of 53 kHz. As the spot size increases, although the overall signal amplitude rises, the energy distribution spans a broader frequency range, leading to the emergence of multiple frequency components and a shift in the center frequency, which ultimately reaches 67 kHz at a spot size of 3.5 mm. This suggests that while a larger spot enhances signal amplitude, it also complicates the frequency response, underscoring the necessity of optimizing laser spot parameters in practical applications to achieve clear and accurate imaging. By systematically varying laser power levels to 80%, 90%, and 100%, with pulse widths of 80 ns and 100 ns, and examining distances of 2 mm and 3.5 mm from the focal point, the system achieved high-resolution imaging that accurately represented the internal state of the batteries. At a laser power of 100% and a pulse width of 80 ns, defect width measurements exhibited an error rate of less than 2%, demonstrating the method’s high precision. However, when the laser incidence distance from the focal point increased to 3.5 mm, a larger dark area emerged around defect sites, with the measurement error reaching 14%, underscoring the need for precise control of laser focus for optimal imaging quality. Based on the attenuation characteristics of ultrasonic signals in different media, laser ultrasonic C-scan imaging was performed on LIB samples. The C-scan results were consistent with the ultrasonic microscope scan results of the gas pore defects in the LIBs, further validating the effectiveness of laser ultrasonic detection for gas defect detection in LIBs. These results highlight the capability of laser ultrasound technology to provide high-resolution, real-time insights into the internal conditions of LIBs, offering a reliable method for enhancing the safety and performance of batteries in various applications.

## Figures and Tables

**Table 1:** The actual, calculated, and error values of the artificial defect width in the LIB sample.

Parameter	$L_{0}$ (mm)	Th (V)	$L_{1}$ (mm)	$L_{2}$ (mm)	$L_{3}$ (mm)	$\bar{L}$ (mm)	ε
80 ns, 80%, focus position	25	0.071	25.5	25	23.5	24.67	1.32%
80 ns, 90%, focus position	25	0.083	26	25	23.5	24.83	0.68%
80 ns, 100%, focus position	25	0.093	25.5	24	24.5	24.67	1.32%
100 ns, 100%, focus position	25	0.108	26	25.5	22.5	24.67	1.32%
80 ns, 100%, 2 mm from the focal point	25	0.089	26.5	28.5	30	28.33	13.32%
80 ns, 100%, 3.5 mm from the focal point	25	0.089	28	27.5	30	28.5	14%

## Data Availability

Data are contained within the article.
